# Bacterial, fungal and parasitic co-infections in leprosy: A scoping review

**DOI:** 10.1371/journal.pntd.0011334

**Published:** 2023-05-22

**Authors:** Luis Alberto Ribeiro Fróes, Tereza Setsuko Toma, Marie Jachiet, Laurie Rousset, Rosana Evangelista Poderoso, Maria Angela Bianconcini Trindade

**Affiliations:** 1 Departamento de Patologia, Faculdade de Medicina, Universidade de São Paulo, São Paulo, SP, Brasil; 2 Núcleo de Evidências, Instituto de Saúde, Secretaria de Estado da Saúde, São Paulo, SP, Brasil; 3 Service de Dermatologie, Hôpital saint Louis APHP Paris, Université Paris Cité; 4 Biblioteca da Faculdade de Ciências Médicas, Universidade de Campinas, SP, Brasil; 5 LIM56, Hospital das Clínicas, Faculdade de Medicina, Universidade de São Paulo, São; Hospital Infantil de Mexico Federico Gomez, MEXICO

## Abstract

**Background:**

In leprosy patients, the most commonly reported non-viral co-infections are Tuberculosis, Leishmaniasis, Chromoblastomycosis and Helminths. The presence of a secondary infection is believed to increase the likelihood of leprosy reactions. The purpose of this review was to describe the clinical and epidemiological characteristics of the most reported bacterial, fungal, and parasitic co-infections in leprosy.

**Methodology/Principal findings:**

Following the PRISMA Extension for Scoping Reviews guidelines, a systematic literature search was conducted by two independent reviewers, resulting in the inclusion of 89 studies. For tuberculosis, a total of 211 cases were identified, with a median age of 36 years and male predominance (82%). Leprosy was the initial infection in 89% of cases, 82% of individuals had multibacillary disease, and 17% developed leprosy reactions. For leishmaniasis, 464 cases were identified, with a median age of 44 years and male predominance (83%). Leprosy was the initial infection in 44% of cases, 76% of individuals presented with multibacillary disease, and 18% developed leprosy reactions. Regarding chromoblastomycosis, we identified 19 cases with a median age of 54 years and male predominance (88%). Leprosy was the primary infection in 66% of cases, 70% of individuals had multibacillary disease, and 35% developed leprosy reactions. Additionally, we found 151 cases of co-infection with leprosy and helminths, with a median age of 43 years and male predominance (68%). Leprosy was the primary infection in 66% of cases, and 76% of individuals presented with multibacillary disease, while the occurrence of leprosy reactions varied from 37% to 81% across studies.

**Conclusion:**

We observed a male-dominated pattern of co-infections among working-age individuals with multibacillary leprosy. Unlike prior studies reporting increased leprosy reactions in chronic viral co-infections, our findings did not indicate any increase among bacterial, fungal, or parasitic co-infections. Rather, co-infections with tuberculosis and leishmaniasis appeared to reduce leprosy reactions.

## Author summary

Our review summarizes the clinical and epidemiological characteristics of non-viral co-infections in leprosy patients, including tuberculosis, leishmaniasis, chromoblastomycosis, and helminths. By conducting a systematic literature search, my colleagues and I identified 89 studies and found a male-dominated pattern of co-infections among working-age individuals with multibacillary leprosy. Our findings suggest that co-infections with tuberculosis and leishmaniasis may reduce the occurrence of leprosy reactions, which is in contrast to prior studies reporting increased reactions in chronic viral co-infections. Additionally, we observed that the occurrence of leprosy reactions varied from 37% to 81% across studies, depending on the type of helminth co-infection, which is higher than the general population. Overall, our study highlights the importance of understanding the potential impact of co-infections on leprosy patients, as it may affect their disease outcomes and treatment. This information is relevant not only to researchers and healthcare providers but also to the general public, as leprosy is still a significant public health problem in many parts of the world. Our review adds to the growing body of knowledge in the field of infectious diseases and underscores the need for further research on this topic.

## 1. Introduction

Leprosy is a neglected tropical disease caused by *Mycobacterium leprae* and *Mycobacterium lepromatosis*, resulting in permanent physical disability. The global incidence of the disease has declined from over 5 million cases in the 1980s to 133,802 cases in 2020, with 140,594 new cases reported in 2021, a 10·2% increase from the previous year [1]. The majority of new cases occur in developing countries, with 94·6% reported in the 23 countries with the highest incidence [2].

Co-infections occur when two or more genetically distinct pathogens are present in the same host. The coexistence of different pathogens can affect the burden of each agent, and the interactions between the pathogens can result in altered clinical manifestations and outcomes in co-infected individuals. Frequently, neglected populations present with multiple co-occurring chronic infectious diseases, mostly characterized by gradual onset and subtle manifestations, which may not elicit complaints. Consequently, diagnosis frequently occurs during later, debilitating stages of the illness, with elevated morbidity rates.

In leprosy, co-infections often involve pathogens prevalent in developing countries and can modify disease progression, increase the risk of leprosy reactions, or affect the efficacy of treatment [3]. Antimicrobial drug interactions between leprosy multidrug therapy and other treatments can also pose challenges [4]. Publications on leprosy co-infections have focused on Tuberculosis, Leishmaniasis, Chromoblastomycosis and Helminthic infections. There is a growing body of evidence on these co-infections, however, there are still many knowledge gaps and a lack of consensus on the topic. We performed a preliminary search on MEDLINE, Cochrane Database of Systematic Reviews and JBI Evidence Synthesis that did not identify any scoping review on bacterial, fungal, and parasitic co-infections in people with leprosy.

In light of the growing and potentially diverse evidence, a scoping review was deemed the most appropriate method to map the clinical and epidemiological characteristics of commonly reported bacterial, fungal, and parasitic co-infections in leprosy and identify knowledge gaps for future research.

## 2. Methods

This review was guided by the Joanna Briggs Institute (JBI) framework for scoping reviews [5] and aimed to address the following question:

*"What are the key clinical and epidemiological features of bacterial, fungal, and parasitic co-infections frequently reported in individuals with leprosy?"*

The objectives of the review were:

- To determine the most prevalent bacterial, fungal, and parasitic co-infections in individuals with leprosy.
- To compile epidemiological information on the topic.
- To characterize the clinical presentations and treatment considerations for co-infected patients.

### 2.1. Search strategy

Between September 20th to 30th, 2022, a librarian conducted searches in the PubMed/MEDLINE, Virtual Health Library—VHL (excluding Medline), Embase, Scopus, and Google Scholar databases. The search strategies were established by incorporating keywords structured using the acronym PCC (Population: individuals with leprosy; Concept: clinical and epidemiological characteristics; Context: co-infections with Tuberculosis, Leishmaniasis, Schistosomiasis, Chagas Disease, fungal and helminthic infections). The complete search strategy can be accessed at https://osf.io/wvcuh/?view_only=b7d8554cc46c4ba2aaecff9cd9e02f50.

### 2.2. Eligibility criteria

This scoping review considered both primary and secondary studies that addressed bacterial, fungal, and parasitic co-infections in patients with leprosy. Articles published in English, Portuguese, Spanish, and French were eligible for inclusion. Studies that analyzed infections independently of leprosy and studies not relevant to the scoping review’s objectives were excluded from consideration.

### 2.3. Source of evidence selection

The process for selecting sources of evidence was based on established eligibility criteria. Following the elimination of duplicates, two individual reviewers independently performed the selection procedure. A bibliographic manager software (Rayyan QCRI) was utilized to evaluate titles and abstracts, and disputes were resolved through consensus or with the assistance of a third reviewer. The articles resulting from the searches and relevant references cited within those articles underwent thorough examination.

### 2.4. Data extraction

The process of data collection involved the creation of a Microsoft Excel spreadsheet. The spreadsheet included details such as: causative pathogen, lead author and year of publication, study methodology, location, sample size, average age, gender, ethnicity, incidence rate, leprosy subtype (multibacillary vs paucibacillary), presence of leprosy reactions, treatment, declaration of potential conflicts of interest, and financial backing. The data extraction was performed by one reviewer, and the accuracy was confirmed by a secondary reviewer.

### 2.5. Analysis of evidence and presentation of results

The results are displayed descriptively through tables, images and graphs for effective presentation. The assessment of the methodological quality of the selected studies was not carried out as it is not pertinent to the outcomes of a scoping review. The results adhered to the guidelines provided by the PRISMA Extension for Scoping Reviews tool [6]. The aims, inclusion criteria and procedures for the scoping review were predefined and documented in a protocol that is accessible at https://osf.io/wvcuh/?view_only=b7d8554cc46c4ba2aaecff9cd9e02f50.

## 3. Results

**Fig 1:**
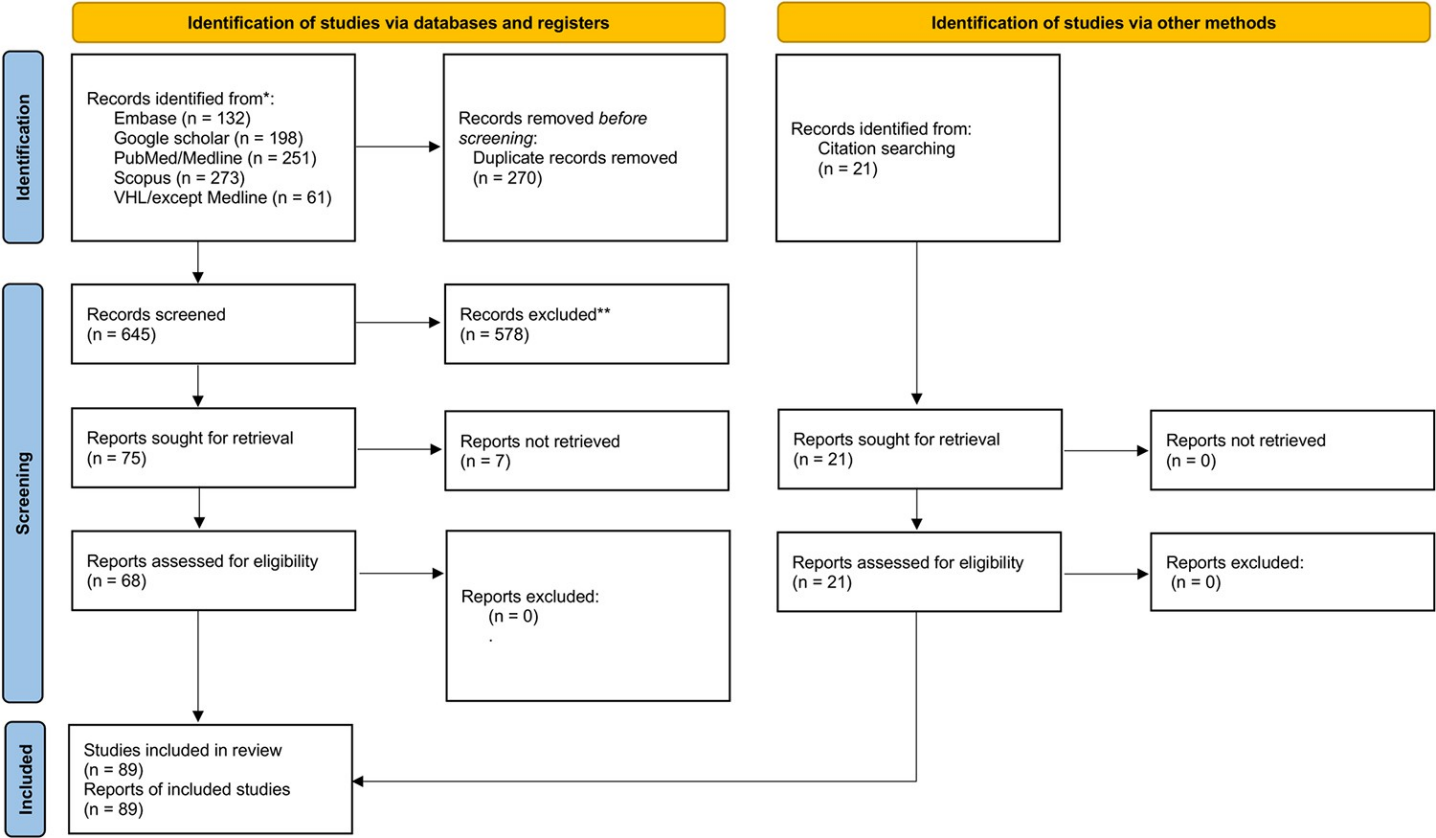
Flow chart of the selection process.

The literature search process generated a total of 645 articles, after duplicates were removed. The remaining articles were screened based on the established eligibility criteria, resulting in the exclusion of 578 articles during the title and abstract screening stage, 75 articles during full-text screening, and 7 reports that could not be obtained. Further, 21 relevant articles were discovered in the reference lists of the included articles, resulting in a total of 89 articles being included in the review. The flowchart of the publication selection process is depicted in Fig 1, following the PRISMA [5] guidelines. The list of excluded studies, along with reasons for exclusion, are available at https://osf.io/wvcuh/?view_only=b7d8554cc46c4ba2aaecff9cd9e02f50.

**Fig 2:**
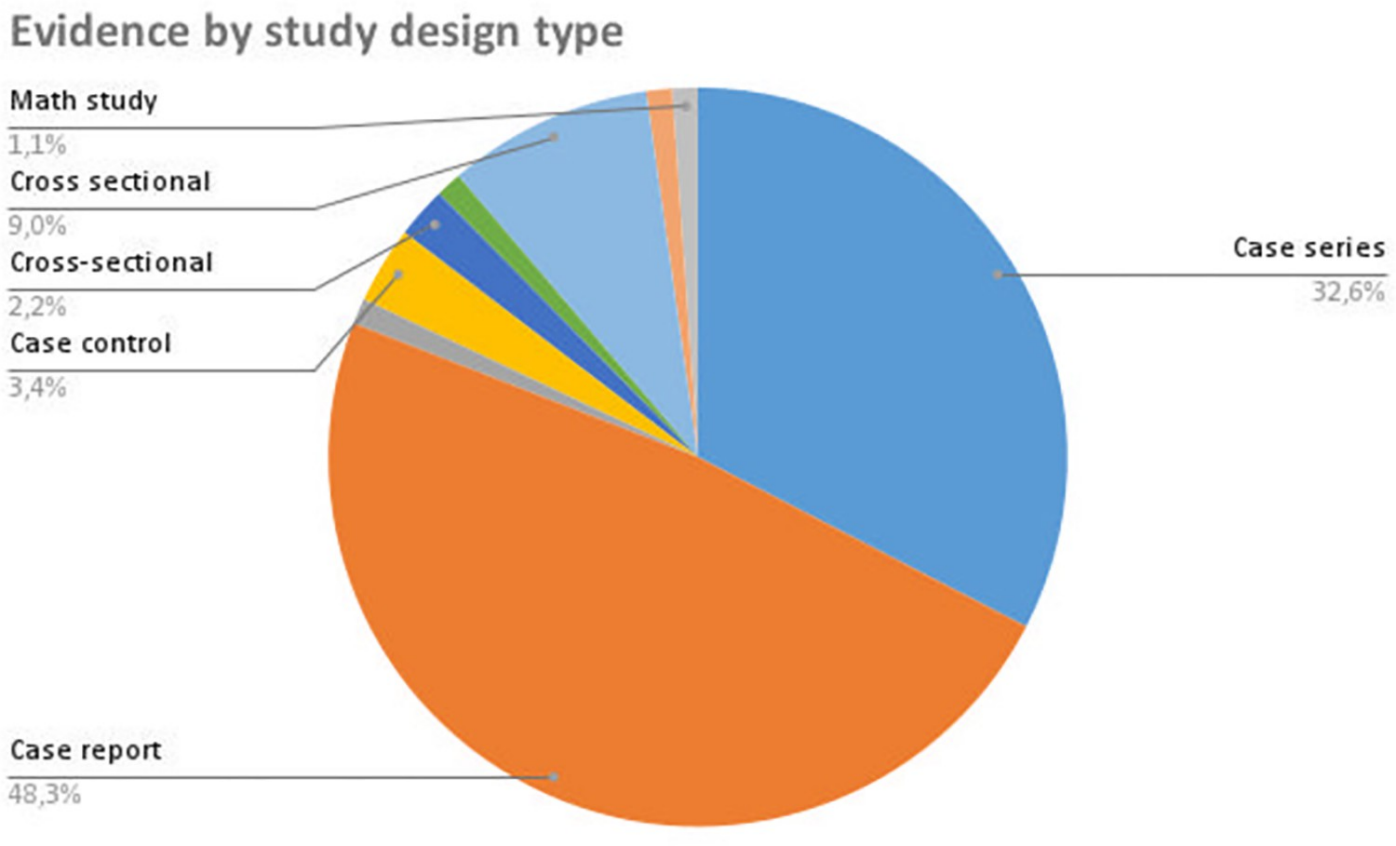
Distribution of the included studies by study design.

**Fig 3:**
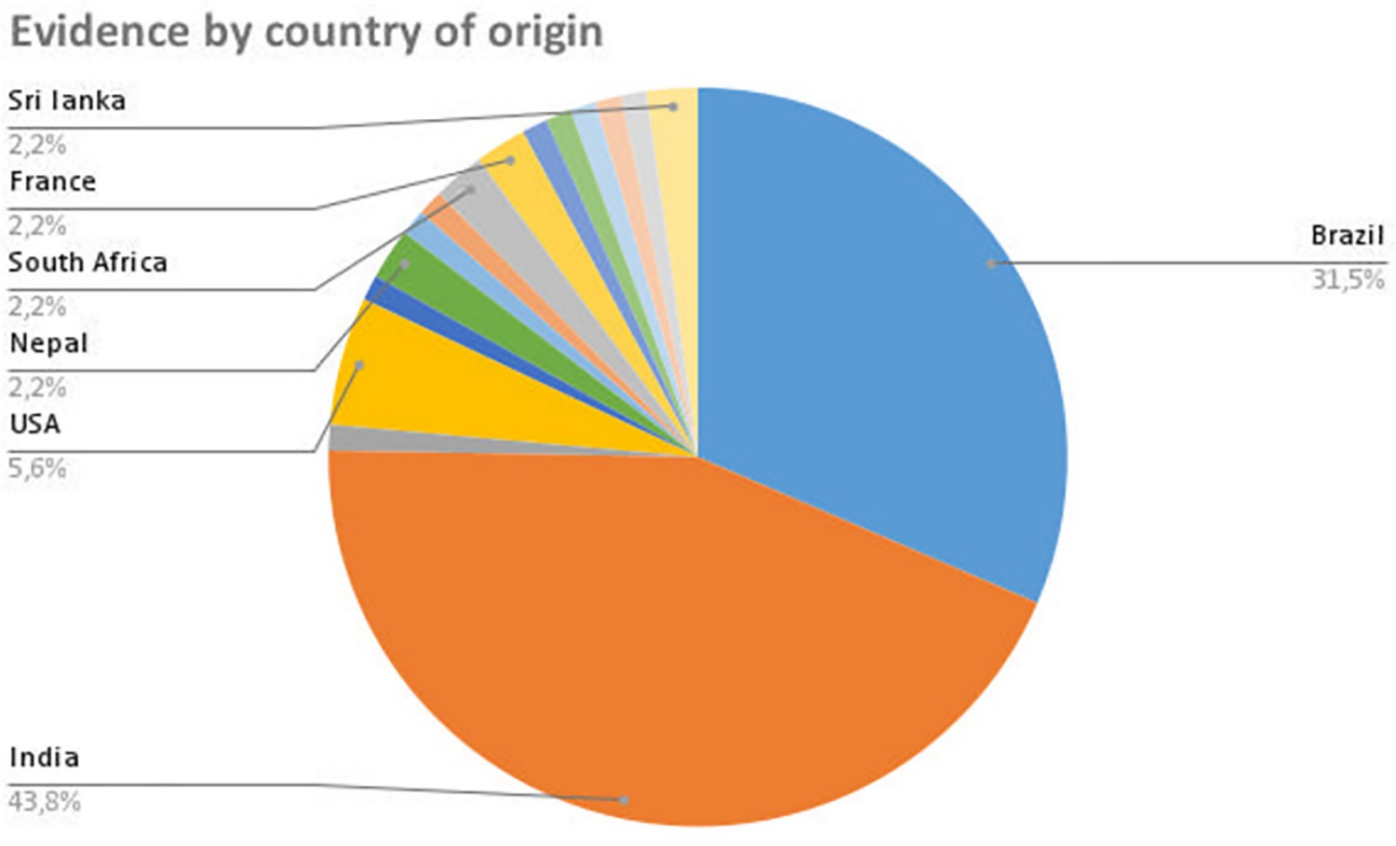
Distribution of the included studies by country.

Our search identified a total of 15 publications related to fungal co-infections, 6 publications related to helminthic co-infections, 14 publications related to protozoal co-infections, and 54 publications related to bacterial co-infections. Of the papers included, 48% were case reports and 32.6% were case series, as shown in **Fig 2**. Among the studies analyzed, 43.8% were conducted in India and 31.5% in Brazil, as illustrated in **Fig 3**. The sample size varied from 1 to 28,204 individuals. We mapped 211 cases of co-infection with leprosy and tuberculosis in the literature, with a median age of 36 years and a male-to-female ratio of 4.7:1. We found 464 cases of co-infection with leprosy and leishmaniasis, with a median age of 44 years and a male-to-female ratio of 4.9:1. In addition, we detected 20 cases of co-infection with leprosy and chromoblastomycosis, with a median age of 53.5 years and a male-to-female ratio of 8.5:1. Other co-infections were reported less frequently and will be discussed further below. Leprosy reactions were reported in 35% of the co-infected cases. Regarding study characteristics, 73% of the papers discussed issues related to treatment, while 45% disclosed financial support and conflict-of-interest statements. Ethnicity of the study participants was reported in only 6.7% of the studies. **Table 1** summarizes the most relevant clinical and epidemiological findings. Outcomes data for each source of evidence are fully available in a spreadsheet table published elsewhere (https://osf.io/wvcuh/?view_only=b7d8554cc46c4ba2aaecff9cd9e02f50).

**Table 1:** Main clinical and epidemiological features of co-infected individuals.

	Tuberculosis	Leishmaniasis	Helminths	Chromoblastomycosis
**Number of cases**	211	464	151	20
**Age (median)**	36 years	44 years	43 years	54 years
**Male (%)**	82%	83%	68%	88%
**Leprosy as first infection (%)**	89%	44%	100%*	66%
**Multibacillary (%)**	82%	76%	76%	70%
**Leprosy reactions (%)**	17%	18%	37%– 81%**	35%

*All studies screened for helminths in individuals with a documented history of leprosy

**See discussion section below

## 4. Discussion

### 4.1. Tuberculosis

Tuberculosis and leprosy are known to infect humans since years 7,000 B.C. [7] and 2,000 B.C. [8], respectively. Archaeological evidence show that the co-infection used to be very common [9], and Hansen himself reported tuberculosis as the major cause of death among leprosy patients in 1895 [10]. Reports of the co-infection in modern literature, however, are much scarcer for less clear reasons. A total of 211 cases were reported between 1954 and 2022 [11–59].

There is a predominance of males, with a male-to-female ratio of 4.7 and a median age of 37 years [18–69 years]. Leprosy precedes coinfection in 89% of cases, tuberculosis in 6%, with a median interval between diagnoses of 12 months [0.5–300 months]. Mycobacterial coinfection is reported to be simultaneous in 5% of cases. In this case, as in many imprecisely published articles, it is difficult to assert with certainty the chronology, although cutaneous lesions seemed to precede the appearance of the general signs attributed to tuberculosis. Leprosy is multibacillary in 82% of cases and paucibacillary in 18% of cases. Type 1 reactions were reported in 8% of the patients and type 2 reactions in 9% of the patients. Tuberculosis is pleuropulmonary in 93.6% of cases, cutaneous-mucous in 3.2% of cases, ganglionary in 1.6% cases, laryngeal in 1% of cases; involvement is less commonly neurological or peritoneal. In most cases, the anti-tuberculosis and anti-leprosy treatment allows improvement of both diseases. Thus, in this coinfection, leprosy is usually multibacillary, more often precedes tuberculosis, which is usually pulmonary, with a very variable time interval between the two.

The exact nature of the relationship between leprosy and tuberculosis has been debated at length in the literature and remains unclear. These are two mycobacterial infections characterized by a granuloma and a spectrum of clinical and histological manifestations that reflect the host’s immune response to the infection. Two theories have been proposed to explain this rare association: cross-immunity and coinfection. The cross-immunity hypothesis suggests that patients with latent tuberculosis infection are protected against leprosy, and conversely, individuals with acquired immunity against leprosy have a lower susceptibility to tuberculosis than the general population. The two infections would be antagonistic with relative protection against the other disease by cross-immunity. This hypothesis has been substantiated by the inverse correlation between the incidence of leprosy and tuberculosis, and the protective effect of the Bacille Calmette-Guérin (BCG) vaccine, albeit partial, against both tuberculosis and leprosy. Genetic studies show that *M*. *leprae* and *M*. *tuberculosis* share 90% of their genomes and cross-immunity has been reported by several studies [60–66]. In addition, in a recent meta-analysis with 326,264 patients, BCG vaccination proved to be the most effective prophylactic intervention for leprosy [67].

The coinfection hypothesis postulates that deficiency of cell-mediated immunity resulting from lepromatous leprosy may enhance the vulnerability and mortality rate of individuals from tuberculosis in areas where both diseases are prevalent. An evolutionary outlook suggests that tuberculosis may have subjected co-infected individuals to negative selection, progressively removing them from the population without transmitting the immunological traits that predispose them to both leprosy and tuberculosis. This hypothesis provides a plausible explanation for the historical decline in leprosy rates, which appears to coincide with a rise in tuberculosis cases in the last century.

It is noteworthy that we observed a relatively low frequency of reactions (17%) in our survey, which is lower than the frequency reported in most studies (>30%) [68]. In cases of multibacillary leprosy, which accounted for more than 80% of our cases, the literature reports a frequency of reactions up to 50% or more [69]. The low frequency of reactions found in our study may be attributed to the fact that leprosy preceded tuberculosis in nearly 90% of the cases. This could mean that the infection by *Mycobacterium tuberculosis* occurred after enough time had passed to prevent any further leprosy reactions, which typically occur up to 3 years after treatment. Nevertheless, a lower frequency would not be expected, but rather equivalent to that of other studies. In this case, if the frequency of reactions is indeed reduced in co-infected individuals, then two hypotheses could be raised: first, the immunological compartmentalization of the two diseases would reduce the interaction of the immune response between them; second, cross-immunity may lead to a better-organized immune response against leprosy, without the disturbances that culminate in reactions. Laboratory studies focused on immunology of the co-infected are crucial to clarify the underlying mechanisms.

It is essential to note that diagnosing tuberculosis in a patient with leprosy using only sputum smear examination is not sufficient since the sputum of patients with leprosy, particularly those with multibacillary disease, may be positive for acid-fast bacilli. Therefore, rapid molecular assays for tuberculosis with culture is warranted [27,70]. The interpretation of Interferon Gamma Release Assays (IGRA), which detect latent tuberculosis infection by measuring interferon production, is challenging in cases of leprosy. It is almost always negative in multibacillary forms of the disease due to anergy, but it can be positive in paucibacillary forms even in the absence of tuberculosis due to cross-reactivity between some common antigens in leprosy and tuberculosis [71,72].

It is recommended to administer concurrent treatment for both diseases in patients diagnosed with co-infection. Rifampicin is utilized monthly in the treatment of leprosy and daily in the treatment of tuberculosis. Hence, daily Rifampicin administration is necessary for individuals with co-infection. Using a lower dosage of Rifampicin to treat *M*. *tuberculosis* can result in drug resistance, highlighting the importance of timely co-infection diagnosis [73].

### 4.2. Helminth infections

Coinfections with *Mycobacterium leprae* and helminths are common in regions where soil-transmitted helminth infections are endemic. There is evidence suggesting that metazoan parasites induce Th2 cell-intrinsic regulation, which raises the question of whether a Th2-skewed immune system would be more susceptible to *M*. *leprae* [74]. A cohort study conducted in Nepal examined 145 patients with leprosy using faecal smear microscopy and multiplex PCR to screen for four types of helminths: *Strongyloides stercoralis*, *Ascaris lumbricoides*, *Ancyclostoma duodenale*, and *Necator americanus*. The results showed that 55% of participants had at least one type of helminth, with *A*. *lumbricoides* being detected in 34%, *S*. *stercoralis* in 18%, *A*. *duodenale* in 17%, and *N*. *americanus* in 5% of cases [75]. In another study from Indonesia, 14% of 81 leprosy patients were found to have helminth infections, with *Trichuris trichiura* detected in 6% and *S*. *stercoralis* in 7% of cases, all of which were multibacillary [76].

A retrospective chart study conducted in Brazil over a period of 10 years found a strong association between intestinal helminth infections and lepromatous leprosy, with an odds ratio of 10.88 and a 95% confidence interval of 4.02–29.4 [77]. The study also found a significant correlation between intestinal helminths and bacilloscopic index (r = 0.982, p < 0.01). Patients with co-infections had decreased levels of IFN-γ, a Th1 cytokine, and increased levels of IL-4 and IL-10, Th2 cytokines, suggesting that co-infection might facilitate the development of disseminated leprosy. Infection by intestinal protozoans, such as *Giardia lamblia* and *Entamoeba spp*., was not significantly different between groups, as immunity to intracellular parasitic protozoans is typically associated with Th1 protective mechanisms, and a Th2-shift would not be expected [77].

In a case-control study, a 7.1% prevalence of *Schistosoma mansoni* or *A*. *lumbricoides* eggs was found in leprosy patients using the Kato Katz test. Multivariate analysis indicated that leprosy patients were more likely to be infected with helminths than household contacts (OR: 8.69; 95% CI: 1.50–50.51) [78]. Another Brazilian study found serologic evidence of helminth infections (*Schistosoma mansoni*, *Strongyloides stercoralis*, *Ascaris lumbricoides*) in 11 out of 73 participants (15%). Notably, no statistically significant association was found between helminth coinfections and either type 1 reaction (OR: 0.85, 95% CI: 0.17–4.17), type 2 reaction (OR: 2.41, 95% CI: 0.29–20.0), or all reactions (OR: 1.36, 95% CI: 0.22–8.33) in patients with leprosy reactions versus those without reactions [79].

The potential relationship between co-infections with helminths and leprosy reactions is currently debated. One study conducted in Nepal demonstrated a statistically significant inverse association between helminth co-infection and leprosy reactions, with 63% of patients without reactions being helminth-positive (p = 0.030), while 65% of patients with reactions were helminth-negative (p = 0.023, 76). This suggests that soil-transmitted helminth co-infections may decrease the risk of developing leprosy reactions. However, another study reported a positive association between helminth co-infections and type 2 reaction [76]. Among 11 individuals with leprosy and helminth infections, 8 had type 2 reactions. It is important to note, however, that these 8 patients had received extended corticosteroid treatment prior to sampling, and 6 had *S*. *stercoralis*, which is known to increase in risk with glucocorticoid administration [80].

Despite ongoing debate about leprosy reactions, there is solid evidence of an increased prevalence of helminth infections among leprosy patients. To address this, we recommend that special attention be given to *Strongyloides stercoralis*, a parasitic organism that is able to complete its life cycle within the human body and can therefore propagate and spread endogenously, particularly in individuals with weakened immune systems. Corticosteroid treatment has been associated with a condition known as hyperinfection, regardless of the dose, duration, or route of administration, and even short courses of treatment (6–17 days) have been shown to increase the risk [81].

In addition, infection with the HTLV-I virus is a significant risk factor for disseminated strongyloidiasis. Patients infected with HTLV-I produce high levels of interferon-gamma, which shifts the immune response away from the Th2 pole that is important for defending against helminths [82]. Disseminated strongyloidiasis can also occur in patients with AIDS, although less frequently than in those with HTLV [83]. Because both HIV and HTLV are known to affect leprosy patients more frequently than the general population [84], we recommend that all newly diagnosed leprosy patients undergo routine stool examinations, and that prophylactic treatment with Ivermectin, at a dose of 200 μg/kg/day for 2 days, be administered to all patients beginning corticosteroid therapy, with repeat doses given after 2 weeks [85].

### 4.3. Leishmaniasis

*Leishmania spp*. and *M*. *leprae* share some characteristics: they are both intracellular organisms that require Th1 cellular immune responses for control. Clinically, Th1 immunity results in tuberculoid leprosy and mucocutaneous leishmaniasis, with localized but severe tissue damage. Th2 humoral response activation, in both leprosy and leishmaniasis, is associated with diffuse presentations: lepromatous leprosy and diffuse cutaneous leishmaniasis. In both cases, diffuse disease is manifested by infiltrated nodules and plaques, high parasitic load, and less intense tissue destruction. Intermediate Th1-Th2 responses lead to borderline leprosy and cutaneous (either localized or disseminated) leishmaniasis [86].

Co-infection of leprosy and leishmaniasis is an infrequent occurrence, even in regions where both diseases are endemic. Historically, only a handful of cases, approximately 34, were documented in the literature, with no indication of a clinical interaction between the two diseases [87–97]. However, recent comprehensive studies carried out in Brazil spanning over a decade, involving a sample of 28,204 leprosy cases and 24,771 American Tegumentary Leishmaniasis cases, identified 414 instances of co-infection [98,99]. These cases demonstrated clinically distinct characteristics in comparison to non-co-infected cases, indicating a noteworthy interaction between the two diseases that contradicts the earlier held belief. We present an outline of the clinical features observed in the 448 co-infected individuals reported in the literature.

The majority of co-infected individuals were male (83%) with a mean age of 44 years. Leishmaniasis is an occupational hazard in certain professions such as farmers, timber exploiters, hunters, and military workers, disproportionately affecting men of working age. The higher mean age among co-infected individuals likely reflects leprosy’s long incubation period. Bacilloscopy was more frequently positive in the co-infected individuals, indicating a tendency towards a Th2 immune response. Most patients were classified as multibacillary (76%) and borderline (56%), reflecting the most common local pattern of leprosy, with no significant difference compared to leprosy-only cases. Leprosy reactions were reported in 18% of cases.

Co-infected individuals had higher odds of developing nerve damage (OR: 1.34; 95% CI: 1.09–1.66) and leprosy reactions (OR: 1.35; 95% CI: 1.04–1.76). Previous research suggests that local and systemic infections can increase the frequency of leprosy reactions and cause more severe nerve damage, potentially through the upregulation of inflammatory markers [3,100,101]. A higher incidence of Grade 2 physical disability, an indicator of late diagnosis, was observed in co-infected individuals (OR: 1.61; 95% CI: 1.07–2.43), indicating that nerve damage in this group could also be influenced by poor living conditions and limited access to healthcare.

Leishmaniasis was the initial diagnosis in 56% of the cases, and of these, 77% developed leprosy within 5 years, suggesting that the two pathogens likely coexisted at some point due to the extended incubation period of *M*. *leprae*. Leprosy reactions were observed in 18% of cases, a rate comparable to that of tuberculosis as discussed earlier. Since the two microorganisms exhibit a low genetic resemblance, cross-immunity cannot be ascribed for the lower-than-expected rate of leprosy reactions. Instead, we hypothesize that immune compartmentalization of the two infections, coupled with a degree of under-reporting of leprosy reactions, might underlie the phenomenon.

Cutaneous leishmaniasis was present in 84% of the co-infected cases, while mucocutaneous leishmaniasis was found in 16%. No instances of the rare diffuse form were observed. The Th1 mucocutaneous form was more frequent in co-infected individuals (OR: 2.29; 95% CI: 1.74–3.00). The development of an exaggerated INF-γ-mediated Th1 response plays a significant role in the formation of potentially disfiguring and destructive lesions in the nasal and oropharyngeal cavities [102]. Studies have shown that different immunological mechanisms may underlie the development of various forms of leishmaniasis and leprosy in patients. For instance, Th1-localized or Th2-anergic diffuse leishmaniasis have been associated with lepromatous leprosy [87,88,103]. Additionally, concomitant cases of Th1-mucocutaneous leishmaniasis and Th2-lepromatous leprosy, as well as post-kala-azar dermal Th2-leishmaniasis and leprosy of varied forms, have been reported [89,96,103–107]. This suggests that there may be compartmentalization of T-cell immunity against *M*. *leprae* and *Leishmania*, which may vary within the same host.

With respect to treatment, the majority of cases examined in our review (93%) indicated the usage of a pentavalent antimonial for the treatment of leishmaniasis in co-infected individuals, and no drug interactions were observed. When both infections are diagnosed concurrently, we advise initiating the treatment for leishmaniasis first due to its shorter duration (3–4 weeks), and potentially to prevent the occurrence of leprosy reactions, which are more frequent in co-infection.

Despite the high endemicity of both diseases in certain regions of Brazil, India, and Nepal, there have been limited reports of leprosy-leishmaniasis co-infection, likely due to insufficient research activity in these areas, rather than a genuine rarity. Therefore, healthcare providers working in these regions should remain vigilant for the possibility of co-infection.

### 4.4. Chromoblastomycosis

Chromoblastomycosis is a persistent granulomatous fungal ailment resulting from the transcutaneous implantation of melanized, dematiaceous fungi. The disease initially manifests as verrucous papules that progress to nodules, tumors, plaques, fibrosis, and lymphedema. Infections are primarily caused by *Fonsecaea spp* and *Cladophialophora spp genera*, and the diagnosis is verified through visualization of characteristic fungal elements termed muriform bodies, with a KOH wet mount or skin biopsy. The disease primarily affects immunocompetent adult males, such as farmers, gardeners, and lumberjacks. The countries most affected include Brazil, China, Madagascar, Mexico, and Venezuela, and the estimated global incidence is about 10,000 cases annually [108].

Despite being common diseases, the incidence of co-infection between chromoblastomycosis and leprosy is rare. Our investigation was only able to identify a total of 20 cases of co-infection over almost six decades, the majority of which were reported in Brazil [108–119] ^108–120^. Epidemiological characteristics of patients co-infected with chromoblastomycosis and leprosy revealed commonalities between the co-infection and each disease separately, including a higher prevalence of multibacillary leprosy and infection by *Fonsecaea pedrosoi* among impoverished adult males working in rural areas with limited access to healthcare.

Brazil accounted for almost 80% of co-infection cases, with a smaller number of cases reported in India and Japan. Of the cases identified, 89% were male, 75% were farmers, and the median age was 53.5 years. Leprosy was diagnosed prior to chromoblastomycosis in 63% of cases, with an average interval of 6 years between diagnoses. When leprosy was diagnosed second, the average time was 19 years, which may be attributed to the long incubation period of leprosy. Leprosy reactions were reported in 21% of cases.

In this review, 55% of co-infected individuals were diagnosed with lepromatous leprosy, 91% were classified as multibacillary, and 45% experienced leprosy reactions (9% had type 1 reaction, and 36% had type 2 reaction). The most frequent clinical presentation of chromoblastomycosis was verrucous (30%) and cicatricial (30%) lesions. In 80% of cases, *F*. *pedrosoi* was identified as the cause of disease, 42% of cases were severe, and 25% of patients had received immunosuppressive therapy with steroids to manage leprosy reactions. Currently, the observed findings in co-infected individuals appear to be comparable to those seen in cases of isolated leprosy or chromoblastomycosis, including with regards to treatment response [68,120]. Further investigations with control groups are necessary to establish definitive conclusions.

### 4.5. Other agents

Three patients previously diagnosed with multibacillary leprosy were identified to have subsequently acquired infections with *Sporothrix schenckii* [93]. The mean age of the patients was 60 years, with two males and one female. All three individuals reported contact with cats. Two of the patients were diagnosed with lymphocutaneous sporotrichosis, and the other was diagnosed with disseminated cutaneous sporotrichosis. The mean interval between the diagnosis of leprosy and sporotrichosis was 9 years. The patients received treatment with either terbinafine or itraconazole, and all achieved complete recovery.

Three cases of co-infection involving *Cryptococcus neoformans* were documented in non-HIV-infected patients who had a previous history of treatment for multibacillary leprosy and were concurrently taking thalidomide (100–300 mg/day) and prednisone (1 mg/kg/day) to manage leprosy type 2 reaction [93]. The average age of the patients was 44 years, with representation from two males and one female. One patient exhibited vegetating lesions at the external auditory canal and unilateral hearing impairment. Another patient developed an ulcerating plaque in the upper thoracic region accompanied by fever and malaise. The third patient presented with subcutaneous nodules and cryptococcal meningitis. The mean interval between the diagnosis of leprosy and cryptococcosis was 2 years. All patients responded favorably to treatment with either fluconazole, itraconazole, and/or amphotericin B.

Lobomycosis is a fungal infection caused by *Lacazia loboi* that affects the skin and subcutaneous tissue, manifesting as chronic keloid-like lesions. The disease is mainly reported in male farmers and rubber workers in the Amazon region of Brazil and Colombia. An 89-year-old man was diagnosed with both lepromatous leprosy and lobomycosis, with the latter presenting as a keloid-like lesion that had persisted for more than three decades [121]. Coinfection with these two diseases is extremely rare, with only a small number of cases reported in the literature. One large retrospective study reported 10 co-infected individuals out of a sample of 249 lobomycosis patients. The study did not provide detailed information about the type of leprosy or the treatments, but it did report that these 10 patients responded best to treatment for lobomycosis, possibly due to a synergistic effect between clofazimine and itraconazole against *Lacazia loboi* [122].

We also report a case of a 56-year-old Afro-Brazilian woman with lepromatous leprosy and recurrent episodes of type 2 reaction who was diagnosed with Chagas disease. The patient developed dyspnea and anasarca after eight months of treatment with rifampicin, dapsone, clofazimine, thalidomide, and prednisone, leading to a diagnosis of class III functional heart failure. Further diagnostic testing confirmed the presence of positive antibodies against *Trypanosoma cruzi* in the patient’s serum. Although the pathogenesis of these two conditions is not directly related, it is possible that the use of corticosteroids may have contributed to the development of heart failure through mechanisms such as worsening hypertension, sodium retention, and ventricular remodeling through effects on the angiotensin receptor [123].

## 5. Conclusion

This scoping review provides a comprehensive overview of the literature pertaining to bacterial, fungal, and parasitic co-infections in leprosy. The review highlights important clinical and epidemiological characteristics of the co-infections reported in the literature. However, the majority of studies identified are limited by their small sample sizes, study designs, and statistical power. Moreover, many studies did not report on leprosy reactions that can take up to three years after treatment to manifest, and the long incubation period for leprosy can make it difficult to determine whether microorganisms co-existed in the host at any point in time. Thus, the definition of co-infection is questionable in some cases. Nonetheless, case reports can still be valuable in revealing differences in immune responses to different organisms.

Ideally, studies should begin with population databases to identify the largest possible number of cases. Our survey identified only two such studies on leprosy-leishmaniasis co-infection [98,99], highlighting the need for further research on co-infections. Researchers residing in endemic areas for any co-infection are encouraged to conduct similar studies for other infections instead of solely reporting cases they encounter in their clinical practice, except when population databases are not available. Additionally, we encourage studies that explore co-infection of leprosy and syphilis, given the near absence of investigations in this domain, despite the exceedingly high prevalence of syphilis. Furthermore, given the substantial migration flow, clinicians in developed countries must be cognizant of neglected diseases, particularly in populations with elevated social vulnerability and immunosuppression, which may result in elevated morbidity, transmission, and disabilities.

Our scoping review aimed to identify gaps in the literature to guide a future systematic review. However, the scarcity of good evidence precludes undertaking such a review at this time. Thus, high-quality research is urgently needed to investigate every aspect of the interaction between *M*. *leprae* and bacteria, fungi, and parasites. Large epidemiological studies are necessary to establish clinical and epidemiological features with statistical significance. Given the low numbers of eligible patients even in specialized centers, setting up such studies will require multi-center collaborations.
